# Treatment of intestinal graft-versus-host disease with unrelated donor fecal microbiota transplantation capsules: A case report

**DOI:** 10.1097/MD.0000000000022129

**Published:** 2020-09-18

**Authors:** Dan Mao, Qi Jiang, Ye Sun, Yubo Mao, Lili Guo, Yanqing Zhang, Muran Man, Guifang Ouyang, Lixia Sheng

**Affiliations:** a Department of Hematology, Ningbo First Hospital; b Internal Medicine, School of Medicine Ningbo University; c Department of Hematology, Ningbo Ninth Hospital, Ningbo, Zhejiang Province, China.

**Keywords:** capsule fecal microbiota transplantation, clinical efficacy, intestinal graft-versus-host disease, intestinal microecology

## Abstract

**Introduction:**

Fecal microbiota transplantation (FMT), administering fecal suspensions via a nasoduodenal tube, has achieved a promising effect in the treatment of intestinal graft-versus-host disease (GvHD) in some pilot studies. In this study, oral FMT capsules from unrelated donor were used for the first time in the treatment of intestinal GvHD. Patient concerns: A 31-year-old male who was diagnosed as “myelodysplastic syndromes with excess blasts II” (intermediate risk 2 of international prognostic scoring system) received human leukocyte antigen -matched sibling donor allogeneic hematopoietic stem cell transplantation. The patient developed diarrhea, vomiting, and bloody stool on 28 days after transplantation.

**Diagnosis:**

Intestinal acute GvHD was diagnosed clinically with histological confirmation by colonoscopy and pathological biopsy.

**Interventions:**

This patient was treated with first cycle of oral FMT capsules after failure to initial treatment of methylprednisolone (2 mg/kg/d) combined with recombinant human tumor necrosis factor–α receptorII: IgG Fc fusion protein (25 mg, biw). The symptoms of intestinal GvHD were relieved but recurred 11 days later. Second cycle of oral FMT capsules was carried out.

**Outcomes:**

After 2 cycles of fecal bacteria transplantation, intestinal GvHD was gradually controlled and did not recur again during the 2-month follow-up. The diversity and structure of the intestinal flora after FMT was closer to that of healthy donors than that before.

**Conclusion:**

Our case showed oral FMT capsules could be used as a treatment option for corticosteroid refractory intestinal GvHD. Further studies are warranted to assess the clinical efficacy and safety of oral FMT capsules in the treatment of intestinal GvHD.

**Rationale:**

Fecal microbiota transplantation (FMT), administering fecal suspensions via a nasoduodenal tube, has achieved a promising effect in the treatment of intestinal graft-versus-host disease (GvHD) in some pilot studies. In this study, oral FMT capsules from unrelated donor were used for the first time in the treatment of intestinal GvHD.

**Patient concerns:**

A 31-year-old male who was diagnosed as “myelodysplastic syndromes with excess blasts II” (intermediate risk 2 of international prognostic scoring system) received human leukocyte antigen -matched sibling donor allogeneic hematopoietic stem cell transplantation. The patient developed diarrhea, vomiting, and bloody stool on 28 days after transplantation.

**Diagnoses:**

Intestinal acute GvHD was diagnosed clinically with histological confirmation by colonoscopy and pathological biopsy.

**Interventions:**

This patient was treated with first cycle of oral FMT capsules after failure to initial treatment of methylprednisolone (2 mg/kg/d) combined with recombinant human tumor necrosis factor–a receptorII: IgG Fc fusion protein (25 mg, biw). The symptoms of intestinal GvHD were relieved but recurred 11 days later. Second cycle of oral FMT capsules was carried out.

**Outcomes:**

After 2 cycles of fecal bacteria transplantation, intestinal GvHD was gradually controlled and did not recur again during the 2-month follow-up. The diversity and structure of the intestinal flora after FMT was closer to that of healthy donors than that before.

Conclusion: Our case showed oral FMT capsules could be used as a treatment option for corticosteroid refractory intestinal GvHD. Further studies are warranted to assess the clinical efficacy and safety of oral FMT capsules in the treatment of intestinal GvHD.

**Lessons:**

There is still a possibility of recurrence after the treatment of GvHD with capsule fecal microbiota transplantation. How to optimize the dosage and treatment course of fecal microbiota capsule administration needs further exploration.

## 1 Introduction

Graft-versus-host disease (GvHD) is a common complication after allogeneic hematopoietic stem cell transplantation, and mainly affects the skin, liver, and intestines. Among these forms, the incidence of intestinal GvHD is high; its degree is serious, the treatment choice is limited, severe intestinal GvHD is often difficult to reverse, and it will cause a series of complications, affecting the outcome of transplantation and increasing the early mortality associated with transplantation. At present, glucocorticoid is the first-line treatment for intestinal GvHD, but nearly half of patients have no response to hormone therapy.^[1]^ There is currently no standard second-line treatment for intestinal GvHD, so patients with intestinal GvHD urgently need more effective treatment.^[2]^ Under normal physiological conditions, highly diverse intestinal microorganisms and related metabolites play an important role in regulating the host intestinal immune balance and nutritional metabolism and in maintaining intestinal mucosal barrier function.^[3]^ Under the condition of allogeneic stem cell transplantation (allo-HSCT), with conditioning regimen-related damage and broad-spectrum antibiotic application, the parasitic microbiota in the gastrointestinal tract and other bacteria-containing organs die and release a large amount of lipopolysaccharides (LPS). The LPS enters the blood circulation through the damaged mucosal barrier and reaches tissues and organs throughout the body. As a secondary signal, LPS can activate lymphoid tissue, digestive tract mononuclear phagocytes, keratinocytes, and fibroblasts related to the intestinal mucosa, leading to the secretion of a large number of inflammatory factors. The resulting inflammatory reaction further expands the local damage, and promotes the occurrence of GvHD.^[4]^ It has been shown that the structure and composition of the intestinal flora changes significantly after transplantation, and the reduction of intestinal flora diversity may play an important role in the development of acute graft-versus-host disease (aGvHD). It can therefore be seen that the imbalance of intestinal microecology is a very important link in the occurrence and development of GvHD. Regulation of the intestinal microecology is becoming a new way to treat GvHD and has achieved preliminary results in some clinical trials. Some studies have shown that infusion of third-party fecal microbiota through a nasoduodenal tube has a certain curative effect in the treatment of intestinal GvHD,^[5]^ but this method involves an invasive operation. In contrast, capsule fecal bacteria transplantation involves liquid fecal bacteria being placed into capsules, which the patient takes orally. The technique has achieved results in the treatment of inflammatory bowel disease and *Clostridium difficile* infection. However, it has not been reported in the treatment of intestinal GvHD. The transplantation of fecal bacteria by oral capsule avoids an invasive operation, and patients have better tolerance of this method. Fecal microbiota transplantation (FMT) is more suitable for patients with GvHD who have low platelet counts and high risk of bleeding after hematopoietic stem cell transplantation. However, there are few studies on the clinical efficacy of capsule fecal microbiota transplantation. In this paper, we report a case of GvHD treated by capsule fecal microbiota transplantation.

## 2 Case presentation

A 31-year-old man was diagnosed with myelodysplastic syndromes with excess blasts II (intermediate risk 2 of international prognostic scoring system, high risk of WHO-based prognostic scoring system) on May 4, 2019. He had human leukocyte antigen (HLA) compatible sibling donors. After excluding secondary factors and transplantation contraindications, the patient underwent HLA-matched sibling donor allogeneic hematopoietic stem cell transplantation. The conditioning regimen consisted of decitabine (15 mg/m^2^/d) for 5 days, ldamycin (10 mg/m^2^/d) for 3 days, cytarabine (4 g/m^2^/d) for 2 days, busulfan (3.2 mg/kg^2^/d) for 3 days, cyclophosphamide (1.8 g/m^2^/d) for 2 days, and semustine (MeCCNU [250 mg/m^2^/d]) for 1 day. GVHD prophylaxis consisted of cyclosporin A, methotrexate, and mycophenolate mofetil. The patient received a total of 6.5×10 ^8^/kg peripheral blood mononuclear cells and 4.2×10^6^/kg CD34+ cells from HLA identical sibling donors. This was followed at+12 days with implantation of granulocytes and megakaryocytes; at +15 day short tandem repeat (STR) indicated complete chimerism of the donor; and at + 22 day he underwent abone marrow puncture and bone marrow routine. The results showed that the 3 cell lines were proliferative and active, the number of primitive cells was not high, the minimal residual disease was negative, and the chimerism analysis using STRs proved fully donor chimeric.

On the 28th day after transplantation, the patient developed diarrhea and vomiting; the amount of diarrhea was about 2000 to 4000 mL/d, and was accompanied by abdominal pain and a congestive skin rash in the palms of both hands. After second-generation sequencing and the detection of pathogenic microorganisms in the stool ruled out infectious diseases, the patient was diagnosed with third-degree intestinal GvHD. After the initial treatment of methylprednisolone (2 mg/kg/d) combined with recombinant human tumor necrosis factor–α receptorII: IgG Fc fusion protein for injection (25 mg, biw), the vomiting and abdominal pain were relieved, but the amount of diarrhea was still more than 2000 mL/d, and bloody stool and jaundice appeared 5 days after the treatment, which was considered to indicate initial treatment failure.

**Figure 1:**
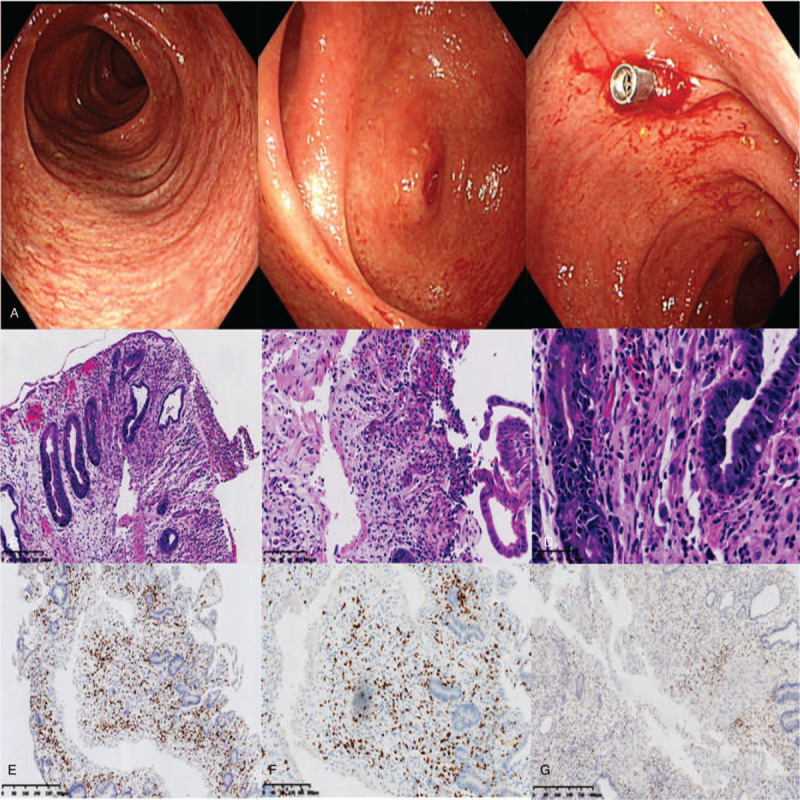
Colonoscopy and pathological findings from the patient. A, Colonoscopy showed congestion and edema of the intestinal mucosa. B–D, Hematoxylin and eosin (HE) staining of colonoscopy biopsy specimen. E, F, Immunohistochemical staining of colonoscopy biopsy specimen.

**Figure 2:**
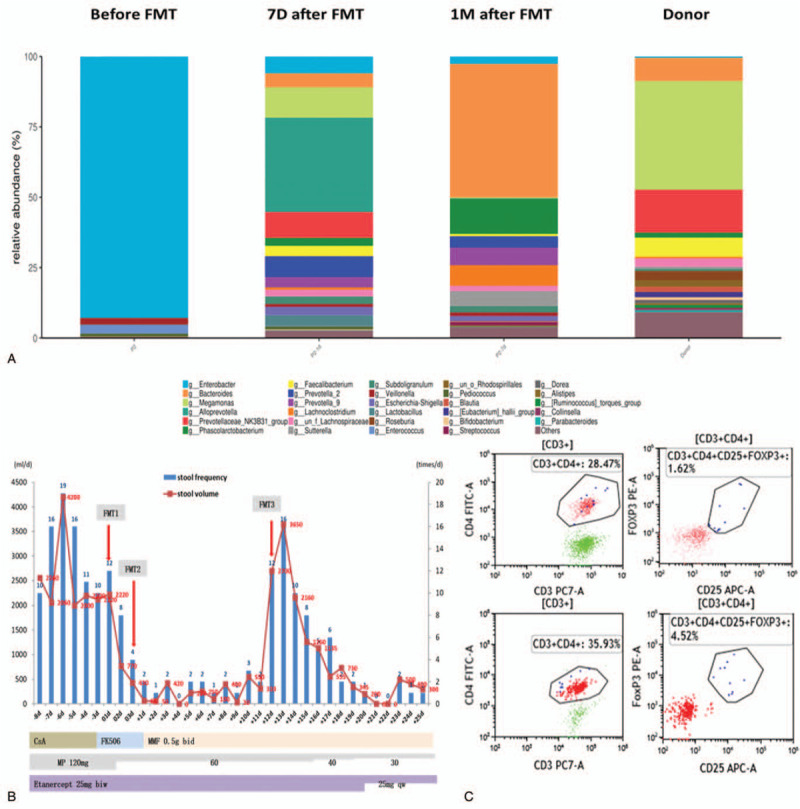
Stool volumes, stool frequency, components of microbiota, and immunological assay before and after FMT. A, Analysis of fecal microbiota composition at the genus level. B, Stool volumes and stool frequency pre and post FMT. C, The ratio of CD4+CD25+FOXP3+ Tregs before FMT and 1 month after FMT. FMT = fecal microbiota transplantation, Tregs = regulatory cells.

During this period, we performed colonoscopy on the patient, and the results showed that the congestion and edema of the terminal small intestinal mucosa were obviously accompanied by erosion, and by congestion and edema of the whole colonic mucosa (Fig. 1A). Histopathology on a biopsy specimen showed mucositis of the small intestine and colon, infiltration of a large number of CD8+T lymphocytes, and no cytomegalovirus inclusion bodies. This result supports the diagnosis of intestinal GvHD (Fig. 1, B–G). After that, we treated the patient with capsular fecal microbiota transplantation (the patient provided informed consent and agreed for publication). After excluding active infection, the antibiotics were stopped 48 hours before transplantation to avoid the influence of antibiotics on the transplanted flora. The specific transplantation method is to take fecal microbiota capsules (provided by the Guangzhou fecal bacteria bank) at a dosage of 30 capsules every other day, for a total of 60 capsules, with each capsule containing the microbial content of 1 g of feces. The patient fasted for 4 hours before and 1 hour after capsule intake, and the capsules should not be crushed, chewed, or dissolved. Stool samples were collected before FMT and 1 week and 1 month after the FMT for genomic 16S ribosomal sequencing. The number and frequency of diarrhea episodes in the patient before and after transplantation are shown in Figure 2B. After the first oral administration of fecal microbiota capsules, the number of episodes of diarrhea and the amount of diarrhea were significantly reduced. Subsequently, the patient took the second dose of fecal microbiota capsule orally. Within 11 days after fecal microbiota transplantation, the number of stools per day remained at 0 to 3. During this period, the amount of diarrhea was significantly reduced compared with that before transplantation, and the symptoms of intestinal GvHD were significantly controlled.

However, 11 days later, the symptoms of intestinal GvHD recurred. We gave 30 fecal microbiota capsules, and following this the intestinal GvHD was gradually controlled. At present, GvHD has not recurred for more than 2 months. We detected 16S rDNA in fecal samples before FMT, 1 week and 1 month after FMT respectively. The results showed that the intestinal microecology of the patient was destroyed before FMT, and that Enterobacteriaceae were dominant. The intestinal microecology after fecal microbiota transplantation, including the diversity and structure of the intestinal flora, was closer to that of healthy donors (Fig. 2A) than that before FMT. At the same time, we found that regulatory cells (Tregs) accounted for 4.52% of the helper T cells in peripheral blood after fecal microbiota transplantation, which was higher than the percentage before fecal microbiota transplantation (Fig. 2C). These results indicated that capsule FMT can treat GvHD by regulating the intestinal microecology, improving the structure of the intestinal flora, reconstructing the diversity of the intestinal microecology, and increasing the proportion of host Tregs.

## 3 Discussion

In recent years, more and more evidence has shown that intestinal micro-organisms play an important role in the development of aGvHD after allo-HSCT. It has been confirmed that a decline of intestinal flora diversity is closely related to the occurrence of aGvHD.^[6,7]^ Tregs have been reported as a predictive cell biomarker of aGvHD.^[8]^ In response to FMT, Tregs increase in number and show terminal differentiation and high inhibition.^[9]^ In addition, Kakihana et al performed transplantation of a fecal microbiological solution on four patients with aGvHD: the outcome was complete remission in 3 patients and partial remission in 1 patient. No adverse effect were found during the trial. The above study shows that FMT has the advantages of high efficiency and safety in the treatment of refractory intestinal GvHD, but it generally uses invasive administration. Compared with traditional microbiological solution transplantation, fecal microbiota capsules can eliminate fungi, parasites, and viruses that may exist in asymptomatic donors, as well as some inflammatory mediators, by optimizing the production process, which can reduce the possibility of disease transmission.^[10]^ Moreover, the administration of oral capsules is easier to expand to multicenter research than nasogastric intubation, enema or endoscope.

As far as we know, this is the first Chinese case of intestinal GvHD in a patient whose symptoms were relieved after treatment with capsule FMT. No significant adverse reactions were found and no infection occurred during the course of fecal bacteria transplantation in the form of capsules and the follow-up period of 3 months. Through 16S rDNA detection of microbial diversity, we found that the diversity of intestinal microflora in the patient was significantly greater after FMT than before, and β diversity analysis also showed that the structure of the intestinal microflora after FMT was close to that of donors, which indicated that the recovery of intestinal microflora diversity could be directly attributed to FMT. After 2 orally administered doses of fecal microflora capsules, the patient's diarrhea was significantly relieved, but 11 days after FMT, the patient had a large number of diarrhea episodes again. This was consistent with cases in the literature: patients receiving microbiological fluid transplantation often show relapse after 1 to 2 weeks.^[2]^ Earlier than us, Kaito et al ^[11]^ reported for the first time that fecal bacteria capsule from sibling donor was successfully used to treat a case of gastrointestinal GvHD patients who failed to respond to the second-line treatment. Consistent with Kaito's report, diarrhea and bloody stool symptoms recurred after the first course of capsule FMT, and the second dose of FMT capsule was still effective, which was consistent with the previously reported experience that repeated dose of FMT brought continuous improvement of gastrointestinal aGvHD symptoms. Compared with siblings as fecal donors, the frozen FMT capsules from unrelated donors we used saved not only cost of production, but also time for waiting, which made FMT more likely to be widely used as off-the-shelf microecological drugs. In addition, the fecal bacteria donor in this case was ruled out of Multidrug-Resistant Organism (MDRO) during the screening period and 1 month after donation, which could avoid the risk of MDRO transmission by FMT. Previous studies have confirmed that an increase of *Blautia* bacteria is related to a decrease of GvHD mortality.^[12]^ Similarly, we found that the number of *Blautia* bacteria in the intestines of the patient after FMT changed from a low level to a normal level, which may be one of the reasons for the improvement of GvHD after FMT. However, the patient still lacks some beneficial bacteria after FMT, and the content of some harmful bacteria is still high, such as *Enterobacter*, *Klebsiella*, etc. In this case, we may consider that the number of probiotic bacteria in the gut of healthy donors is insufficient for patients with GvHD; we may confirm this hypothesis by slightly increasing the proportion of probiotics in the capsule preparation process. It has also been pointed out that giving patients broad-spectrum antibiotics before FMT, to inhibit harmful microorganisms that originally existed in the patients’ intestines, and not using broad-spectrum antibiotics after FMT, will increase the possibility of long-term fecal microflora implantation.^[13]^ If patients need to use broad-spectrum antibiotics due to infection in other parts of the body, the optimum time to restart antibiotics after capsule FMT is worthy of discussion.

The successful experience of this case of the use of capsule FMT in the treatment of intestinal GvHD may provide a basis for a follow-up study of capsule FMT in the treatment of GvHD. However, the efficacy and safety of the capsule form of FMT in the treatment of intestinal GVHD need further study, and the most appropriate dosage and course of treatment need to be optimized.

## 4 Conclusions

As far as we know, this is the first successful case of intestinal GvHD treated by microbiota transplantation using unrelated donor frozen FMT capsules. However, in a single case, it is impossible to determine the efficacy of this form of FMT. Therefore, more clinical research is needed to explore the clinical efficacy and safety of the capsule form of FMT in the treatment of intestinal GvHD, as well as its advantages and disadvantages compared with traditional bacterial fluid transplantation.

## Author contributions

**Conceptualization:** Dan Mao, Qi Jiang, Lixia Sheng, Guifang Ouyang.

**Data curation:** Dan Mao, Qi Jiang, Ye Sun.

**Formal analysis:** Dan Mao, Qi Jiang, Ye Sun, Lixia Sheng, Guifang Ouyang.

**Funding acquisition:** Lixia Sheng, Guifang Ouyang.

**Investigation:** Dan Mao, Yubo Mao, Lili Guo, Muran Man, Yanqing Zhang.

**Software:** Dan Mao, Lixia Sheng, Qi Jiang, Ye Sun.

**Supervision:** Lixia Sheng, Guifang Ouyang.

**Validation:** Dan Mao, Lixia Sheng, Qi Jiang, Ye Sun.

**Visualization:** Dan Mao.

**Writing – original draft:** Dan Mao, Qi Jiang.

**Writing – review & editing:** Dan Mao, Qi Jiang, Ye Sun, Lixia Sheng, Guifang Ouyang.

## Abbreviations

Abbreviations: aGvHD = acute graft-versus-host disease, allo-HSCT = allogeneic stem cell transplantation, FMT = fecal microbiota transplantation, GvHD = graft-versus-host disease, HLA = human leukocyte antigen, LPS = lipopolysaccharides, MDRO = multidrug-resistant organism, STR = short tandem repeat, Tregs = regulatory cells.
